# Bilateral valvular involvement in infective endocarditis: A clinically distinct entity? A multicenter retrospective study

**DOI:** 10.1371/journal.pone.0336847

**Published:** 2025-11-21

**Authors:** Ruchi Bhandari, Nathan L. Maris, Rowida Mohamed

**Affiliations:** 1 West Virginia University School of Public Health, Morgantown, West Virginia, United States of America; 2 Yale University School of Medicine, New Haven, Connecticut, United States of America; 3 University of Washington School of Medicine, Seattle, Washington, United States of America; 4 Pritzker School of Medicine, University of Chicago, Chicago, Illinois, United States of America; Johns Hopkins Medicine, UNITED STATES OF AMERICA

## Abstract

**Background:**

Infective endocarditis (IE), an infection of the cardiac endothelium, is associated with significant morbidity and mortality. IE cases vary substantially based on their valvular involvement, both in terms of risk factors and severity. This study describes patient characteristics and outcomes associated with valvular involvement in IE.

**Methods:**

Data for this multicenter, retrospective study were obtained from electronic medical chart review of adult patients hospitalized for their index admission for IE between January 2014- January 2018 in the Appalachian region, USA. Descriptive summary statistics are presented by valvular involvement: (1) Left-sided Infective Endocarditis (LSIE): aortic/mitral valve; (2) Right-sided Infective Endocarditis (RSIE): tricuspid/pulmonary valve; and (3) Bilateral Infective Endocarditis (BLIE): right and left-sided. Multivariable logistic regression analysis examined the association of valvular involvement with intensive care unit (ICU) admission.

**Results:**

Overall, 726 patients with IE were included in this retrospective study that provides a novel analysis of IE valvular involvement, highlighting how patient characteristics, comorbidities, substance use behaviors, and clinical outcomes differ across LSIE, RSIE, and BLIE presentations. Drug use, the presence of one or two comorbidities, and bilateral or left-sided valve involvement were independently associated with increased odds of ICU admission. Notably, patients with BLIE experienced a greater clinical burden, requiring more frequent specialist consultations, increased use of pain management services, higher mortality, and a higher rate of surgical interventions.

**Conclusion:**

BLIE represents a clinically distinct IE subgroup characterized by dual vulnerabilities: high-risk substance use behaviors associated with RSIE, and medical complexity typically seen in LSIE. Our findings underscore the importance of including BLIE as a distinct category in future epidemiological and clinical investigations of endocarditis valvular involvement.

## Introduction

Infective endocarditis (IE), a bacterial or fungal infection of the cardiac endothelium, is a serious condition associated with significant morbidity and mortality, whose incidence in the United States and worldwide has been increasing in recent decades [1–3]. Much of this rise, particularly in the developed economies, has been attributed to wider implementation of permanent intravascular medical devices, higher prevalence of prosthetic valves, improved survival of patients with congenital heart disease, and, more concerningly, growing intravenous drug use within the broader context of the global opioid crisis [1,4–7].

Cases of IE can be subdivided into three categories based upon their valvular involvement: left-sided IE (LSIE) – infection of the aortic and/or mitral valve, right-sided IE (RSIE) – infection of the pulmonic and/or tricuspid valve, and bilateral IE (BLIE) – infection of at least one valve on each side of the heart. LSIE has historically been the most common manifestation of IE for unclear reasons, though higher transvalvular pressure gradients, increased blood oxygen content, and greater wall stress in the left ventricle have all been proposed as potential mechanisms facilitating intracardiac bacterial growth [8]. This category of IE also tends towards a greater degree of invasiveness and higher patient mortality [9,10]. Streptococcal species have most commonly been implicated as causative organisms in LSIE, though incidence of staphylococcal infections in this subgroup has risen [11].

RSIE, accounting for only 5–10% of all IE cases, has classically been associated with intravenous drug use, as the tricuspid and pulmonic valves are theoretically the first intracardiac structures contacting injected foreign contaminants from peripheral tissues [12,13]. However, surveys of people who inject drugs are less conclusive, suggesting that lesion trends can vary based on study population and even choice of injected substance [14–16]. Regarding its severity, RSIE is typically more indolent than LSIE and can often be managed successfully with antibiotics alone. *Staphylococcus aureus* is usually the causative organism in right-sided infections [8].

Less is known about the characteristics of BLIE, which is frequently grouped with LSIE in analyses or omitted from studies altogether [17,18]. Like LSIE, BLIE appears to pose a greater threat of morbidity and mortality than right-sided disease [17]. Early work also suggests an association between the presence of an intracardiac shunt and BLIE [19]. There is no clear consensus about the predominant organisms that cause BLIE. A recent systematic review with meta-analysis demonstrated that compared to single-valve IE, patients with multivalvular IE had increased risk of short-term mortality by 29%, one-year mortality by 20%, heart failure by 31%, and 22% for subsequent surgical intervention [20]. To our knowledge, there have been no recent, large-scale epidemiological reports on valvular involvement of lesion that specifically include cases with bilateral disease. Given these gaps in knowledge, the purpose of our study is to examine the links between patient characteristics, singular versus bilateral valvular involvement, and outcomes among adult patients hospitalized for infective endocarditis.

## Methods

Data for this multicentre, retrospective study were obtained from a database of patients with IE previously described [7]. All adult patients who were hospitalized for their index admission for IE at four large university-affiliated referral hospitals in the Appalachian region between January 1, 2014 and December 31, 2018 were included in the study. Patients who had an IE diagnosis prior to the study period were excluded from the cohort. This study only includes patients for whom information on the valvular involvement of IE was available. The database was developed from the electronic medical chart review of adult patients identified by IE-associated International Classification of Diseases-10 (ICD-10) codes followed by manual chart review for confirmed IE diagnosis. Data from the manual review were abstracted into a standardized, coded dataset in a secure, HIPAA-compliant, web-based data capture system, Research Electronic Data Capture. Detailed methodology is available elsewhere [7]. The study was approved by the Institutional Review Board at West Virginia University (IRB protocol number: 1811373348). The ethics committee waived the requirement for informed consent. Data were accessed from September 1, 2019 to August 31, 2022.

Descriptive summary statistics are presented as counts and percentages for patient groups stratified by valvular involvement: (1) Left-sided Infective Endocarditis (LSIE): aortic or mitral valve; (2) Right-sided Infective Endocarditis (RSIE): tricuspid or pulmonary valve; and (3) Bilateral Infective Endocarditis (BLIE): right and left-sided. Characteristics are presented on demographics; substance use, including types of drugs; number of comorbidities, including specific somatic comorbidities and psychiatric disorders; causative organisms; specific consultations; length of intensive care unit (ICU) stay; readmission (during the study period); and discharge status. Drug use was defined as any documented use of illicit substances recorded in the medical record prior to hospital admission, regardless of frequency or duration. Substance Use Disorder (SUD) was defined as a formally diagnosed disorder documented in the patient’s electronic health record and categorized under psychiatric comorbidities. Because not all patients with a history of drug use receive an SUD diagnosis, the prevalence of SUD is expected to be lower than that of documented drug use. Characteristics are compared using Chi-square test (or Fisher’s exact test when expected cell count was less than five), with significance level set at *p* < 0.05.

The outcome variable is hospitalized endocarditis patients who were admitted to the ICU compared to patients who were not admitted to ICU, as ICU utilization is an appropriate proxy for high healthcare utilization and patient morbidity. Key predictor variable was valvular involvement (LSIE, RSIE, BLIE). Potential confounders were age (continuous), drug use (yes/no), organism (Methicillin-resistant *Staphylococcus aureus-*MRSA/Methicillin-susceptible *Staphylococcus aureus-*MSSA or other), and comorbidities (zero, one, two, or greater than or equal to three). Multivariable logistic regression analysis was conducted to examine whether an IE patient’s admission to ICU was associated with valvular involvement, adjusting for potential confounders. Results are presented as Odds Ratios (OR) with 95% confidence interval (CI). Statistical analyses were conducted using SPSS version 29.0.

## Results

Overall, 726 patients with IE were included in this study with information on valvular involvement. Characteristics are presented by valvular involvement in the following tables: demographic characteristics in Table 1, substance use characteristics in Table 2, clinical characteristics in Table 3, consultations in Table 4, and hospital utilization characteristics in Table 5. Compared to patients with LSIE, a higher proportion with RSIE and BLIE were: (1) aged between 18 and 44 (LSIE 44.3%, RSIE 81.6%, BLIE 87.5%), (2) female (LSIE 41.7%, RSIE 59.5%, BLIE 58.3%), (3) with a history of drug use (LSIE 53.7%, RSIE 89.9%, BLIE 89.4%), (4) had documented substance use disorder (LSIE 21.3%, RSIE 37.7%, BLIE 43.8%), (5) had MRSA/MSSA (LSIE 51.5%, RSIE 85.0%, BLIE 81.3%), and (6) were discharged against medical advice (LSIE 5.6%, RSIE 22.4%, BLIE 20.8%). All p-values were <0.001.

**Table 1:** Demographic Characteristics of Hospitalized Patients with IE*.

	Bilateral IE*		Right-sided IE*		Left-sided IE*		P-value
	**Count**	**Percent**	**Count**	**Percent**	**Count**	**Percent**	
**Sex**							<.001
Male	20	41.7%	130	40.5%	208	58.3%	
Female	28	58.3%	191	59.5%	149	41.7%	
**Age**							<.001
18-44	42	87.5%	261	81.6%	158	44.3%	
45-64	_	_	49	15.3%	112	31.4%	
≥ 65	_	_	10	3.1%	87	24.4%	

*IE: Infective Endocarditis

Data suppressed for cells without value due to small sample size.

**Table 2:** Substance Use Characteristics of Hospitalized Patients with IE*.

	Bilateral IE*		Right-sided IE*		Left-sided IE*		P-value
	**Count**	**Percent**	**Count**	**Percent**	**Count**	**Percent**	
**Alcohol**							0.413**
Current Use	15	32.6%	59	24.5%	70	23.2%	
Former Use	_	_	31	12.9%	29	9.6%	
No Use	_	_	151	62.7%	203	67.2%	
**Drug Use**							<.001
Yes	42	89.4%	285	89.9%	189	53.7%	
No	5	10.6%	32	10.1%	163	46.3%	
**Buprenorphine**							0.013
Yes	15	31.3%	63	19.6%	53	14.8%	
No	33	68.8%	258	80.4%	304	85.2%	
**Cannabinoids (Marijuana)**							0.003
Yes	14	29.2%	91	28.3%	60	16.8%	
No	34	70.8%	230	71.7%	297	83.2%	
**Opiates**							<.001
Yes	37	77.1%	256	79.8%	152	42.6%	
No	11	22.9%	65	20.2%	205	57.4%	
**Benzodiazepines**							<.001
Yes	9	18.8%	55	17.1%	24	6.7%	
No	39	81.3%	266	82.9%	333	93.3%	
**Cocaine Metabolites**							0.001
Yes	13	27.1%	74	23.1%	44	12.3%	
No	35	72.9%	247	76.9%	313	87.7%	
**Amphetamines**							<.001
Yes	20	41.7%	108	33.6%	45	12.6%	
No	28	58.3%	213	66.4%	312	87.4%	
**MOUD**^**#**^ **Prior to Hospitalization**							<.001
Yes	20	50.0%	86	32.5%	57	20.2%	
No	20	50.0%	179	67.5%	225	79.8%	
**MOUD**^**#**^ **During Hospitalization**							<.001
Yes	14	29.8%	87	27.4%	45	12.7%	
No	33	70.2%	230	72.6%	310	87.3%	

*IE: Infective Endocarditis

**P-value was calculated using Fisher’s exact test

– Data suppressed for cells without value due to small sample size

# MOUD: Medication for Opioid Use Disorder

**Table 3:** Clinical Characteristics of Hospitalized Patients with IE*.

	Bilateral IE*		Right-sided IE*		Left-sided IE*		P-value
	**Count**	**Percent**	**Count**	**Percent**	**Count**	**Percent**	
**Comorbidities**							<.001
0	9	18.8%	102	31.8%	62	17.4%	
1	22	45.8%	141	43.9%	92	25.8%	
2	6	12.5%	50	15.6%	75	21.0%	
≥ 3	11	22.9%	28	8.7%	128	35.9%	
**Stroke**							<.001**
Yes	_	_	8	2.5%	50	14.0%	
No	_	_	313	97.5%	307	86.0%	
**Coronary Artery Disease**							<.001
Yes	_	_	12	3.7%	70	19.6%	
No	_	_	309	96.3%	287	80.4%	
**Type 2 Diabetes**							<.001
Yes	6	12.5%	16	5.0%	89	24.9%	
No	42	87.5%	305	95.0%	268	75.1%	
**Hypertension**							<.001
Yes	8	16.7%	46	14.3%	162	45.4%	
No	40	83.3%	275	85.7%	195	54.6%	
**Hyperlipidemia**							<.001**
Yes	_	_	13	4.0%	58	16.2%	
No	_	_	308	96.0%	299	83.8%	
**Chronic Lung Disease**							0.001
Yes	_	_	22	6.9%	57	16.0%	
No	_	_	299	93.1%	300	84.0%	
**Peripheral Vascular Disease**							<.001**
Yes	_	_	8	2.5%	45	12.6%	
No	_	_	313	97.5%	312	87.4%	
**Chronic Kidney Disease**							<.001**
Yes	_	_	12	3.7%	49	13.7%	
No	_	_	309	96.3%	308	86.3%	
**Acute Kidney Injury**							<.339
Yes	6	12.5%	37	11.5%	30	8.4%	
No	42	87.5%	284	88.5%	327	91.6%	
**Psychiatric Disorders** ^**#**^							0.007
0	23	47.9%	170	53.0%	233	65.3%	
1	10	20.8%	61	19.0%	61	17.1%	
2	5	10.4%	42	13.1%	34	9.5%	
≥ 3	10	20.8%	48	15.0%	29	8.1%	
**Substance Use Disorder**							<.001
Yes	21	43.8%	121	37.7%	76	21.3%	
No	27	56.3%	200	62.3%	281	78.7%	
Type of IE*							<.001
Native valve	–	–	304	96.2%	311	87.4%	
Prosthetic valve	_	_	12	3.8%	45	12.6%	
**MRSA or MSSA** ^**##**^							<.001
Yes	39	81.3%	273	85.0%	184	51.5%	
No	9	18.8%	48	15.0%	173	48.5%	
**Candida Fungi**							0.390
Yes	5	10.4%	18	5.6%	22	6.2%	
No	43	89.6%	303	94.4%	335	93.8%	

*IE: Infective Endocarditis

**P-value was calculated using Fisher’s exact test

– Data suppressed for cells without value due to small sample size

# Psychiatric disorders include Substance use disorder, Depression, Anxiety, Bipolar disorder, PTSD, and Other

## MRSA: Methicillin-resistant *Staphylococcus aureus*; MSSA: Methicillin-susceptible *Staphylococcus aureus*

**Table 4:** Consultation Services Utilized by Hospitalized Patients with IE*.

	Bilateral IE*		Right-sided IE*		Left-sided IE*		P-value
	**Count**	**Percent**	**Count**	**Percent**	**Count**	**Percent**	
**Consultation**							<.007
<=3	10	20.8%	98	30.5%	76	21.3%	
4–6	13	27.1%	122	38.0%	141	39.5%	
>=7	25	52.1%	101	31.5%	140	39.2%	
**Cardiac Surgery**							<.010
Yes	40	83.3%	290	90.3%	294	82.4%	
No	8	16.7%	31	9.7%	63	17.6%	
**Neurology**							<.001
Yes	9	18.8%	16	5.0%	99	27.7%	
No	39	81.3%	305	95.0%	258	72.3%	
**General Surgery**							0.045
Yes	13	27.1%	47	14.6%	72	20.2%	
No	35	72.9%	274	85.4%	285	79.8%	
**Neurosurgery**							<0.001
Yes			16	5.0%	49	13.7%	
No	45	93.8%	305	95.0%	308	86.3%	
**Vascular**							0.003
Yes	8	16.7%	33	10.3%	70	19.6%	
No	40	83.3%	288	89.7%	287	80.4%	
**Dentistry**							0.067
Yes	18	37.5%	86	26.8%	81	22.7%	
No	30	62.5%	235	73.2%	276	77.3%	
**Psychiatry**							<.001
Yes	25	52.1%	152	47.4%	91	25.5%	
No	23	47.9%	169	52.6%	266	74.5%	
**Social work**							0.012
Yes	29	60.4%	141	43.9%	192	53.8%	
No	19	39.6%	180	56.1%	165	46.2%	
**Physical/Occupational Therapy**							<.001
Yes	25	52.1%	109	34.0%	173	48.5%	
No	23	47.9%	212	66.0%	184	51.5%	
**Pain Management**							<.001
Yes	14	29.2%	40	12.5%	16	4.5%	
No	34	70.8%	281	87.5%	341	95.5%	
**Spiritual Counseling**							0.005
Yes	17	35.4%	58	18.1%	94	26.3%	
No	31	64.6%	263	81.9%	263	73.7%	

*IE: Infective Endocarditis

**Table 5:** Hospital Utilization Characteristics of Hospitalized Patients with IE*.

	Bilateral IE		Right-sided IE		Left-sided IE		P-value
	**Count**	**Percent**	**Count**	**Percent**	**Count**	**Percent**	
**Surgery**							0.011
Yes	28	58.3%	116	36.1%	148	41.6%	
No	20	41.7%	205	63.9%	208	58.4%	
**Intensive Care Unit**							0.002
Yes	34	70.8%	167	52.0%	227	63.6%	
No	14	29.2%	154	48.0%	130	36.4%	
**Discharge Status**							<.001
Discharge Alive	31	64.6%	231	72.0%	292	81.8%	
Discharge AMA**	10	20.8%	72	22.4%	20	5.6%	
Death	7	14.6%	18	5.6%	45	12.6%	
**Readmission**							0.225
Yes	9	18.8%	54	16.8%	45	12.6%	
No	39	81.3%	267	83.2%	312	87.4%	
							

*IE: Infective Endocarditis

**AMA: Against Medical Advice

– Data suppressed for cells without value due to small sample size

However, all three groups differed significantly in the proportion of patients with three or more comorbidities (BLIE 23%, LSIE 36%, RSIE 9%), who were on medication for opioid use disorder prior to hospitalization (BLIE 50%, LSIE 32%, RSIE 32.5%), who had psychiatric disorders (BLIE 52%, RSIE 48%, LSIE 35%), and who underwent surgery (BLIE 58%, LSIE 42%, RSIE 36%). Although the number of patients with BLIE was smaller, the proportion of patients who had the most (seven or more) consultations was highest for them (BLIE 52.1%, LSIE 39.2%, RSIE 31.5%,). Similarly, the proportion of in-hospital mortality was also the highest among patients with BLIE (BLIE 14.6%, LSIE 12.6%, RSIE 5.6%).

Results from the multivariable logistic regression analysis are presented in Table 6. After adjusting for confounders, the odds of being admitted to ICU were higher for patients with (1) BLIE (OR: 2.34; 95% CI: 1.174–4.679) and LSIE (OR: 2.36; 95% CI: 1.607–3.468) compared to patients with RSIE, (2) drug use (OR: 2.12; 95% CI: 1.287–3.493) compared to those without drug use, and (3) one comorbidity (OR: 1.83; 95% CI: 1.212–2.771) and two comorbidities (OR: 1.95; 95% CI: 1.170–3.234) compared to patients with no comorbidity. Patients with ≥3 comorbidities also had higher odds of ICU admission, but the estimate was not significant. Age also did not have a significant association.

**Table 6:** Logistic Regression of Admission to Intensive Care Unit for patients with IE*.

	Odds Ratios	95% Confidence Interval		p-value
		**Lower**	**Upper**	
**Age**	0.992	0.978	1.007	0.287
**Drug Use**				
No	Reference			
Yes	2.125	1.287	3.493	0.003
**MRSA or MSSA** ^**#**^				
No	Reference			
Yes	0.977	0.681	1.401	0.898
**Comorbidities**				
0	Reference			
1	1.832	1.212	2.771	0.004
2	1.945	1.170	3.234	0.010
≥ 3	1.493	0.876	2.545	0.141
**Valve**				
Right-sided IE*	Reference			
Left-sided IE*	2.361	1.607	3.468	<.001
Bilateral IE*	2.343	1.174	4.679	0.016

*IE: Infective Endocarditis

# MRSA: Methicillin-resistant *Staphylococcus aureus*; MSSA: Methicillin-susceptible *Staphylococcus aureus*

## Discussion

This retrospective study provides a novel analysis of IE valvular involvement, highlighting how patient characteristics, comorbidities, substance use behaviors, and clinical outcomes differ across LSIE, RSIE, and BLIE presentations. Drug use, the presence of one or two comorbidities, and BLIE or LSIE were independently associated with increased odds of ICU admission.

Consistent with previous research, we found that RSIE primarily affects younger individuals with a history of intravenous drug use [8,16,21]. In contrast, LSIE is associated with older age, higher comorbidity burden, and increased mortality risk [9,10,21]. Patients with RSIE having higher rates of substance use align with the classical thought that injection of foreign contaminants into the venous circulation predisposes them to preferential inoculation of the tricuspid and pulmonic valves [9] In contrast, LSIE seems to have a preference for patients with structural heart disease, prosthetic valves, or chronic illnesses such as diabetes or renal insufficiency, all of which increase the risk of poor outcomes [22,23].

Patients with BLIE, however, displayed a unique hybrid profile. Demographically, they resembled patients with RSIE—primarily young, female, and with high rates of polysubstance use and *Staphylococcus aureus* infection [21]. Our results with a higher proportion of females having BLIE differ from another comparable study where the authors report a lower proportion of women with BLIE [15] Nevertheless, clinically, patients with BLIE mirrored patients with LSIE in terms of somatic comorbidity burden and increased rate of mortality.

Psychiatric comorbidities—such as depression, anxiety, and post-traumatic stress disorder—were significantly more prevalent among patients with RSIE and BLIE. These conditions may exacerbate clinical complexity by influencing adherence to treatment, engagement in substance use recovery programs, and overall prognosis. Clinically, this highlights the importance of integrating mental health screening and support into standard IE management, ensuring that psychiatric care is coordinated alongside infectious disease and addiction services to improve patient outcomes and reduce recurrence risk.

In the multivariable regression analysis, age was not significantly associated with ICU admission. This finding aligns with prior studies showing that the effect of age often becomes nonsignificant when stronger clinical or behavioral predictors are included in multivariable models [24,25]. This suggests that, within high-risk populations such as patients with drug use–associated IE, the impact of age may be attenuated by the stronger effects of comorbidities and other clinical determinants.

In the multivariable analysis, we also observed that odds of ICU admission were higher for patients with one or more comorbidities compared to those with no comorbidity. While higher comorbidity burden is typically associated with worse outcomes, several studies suggest a paradoxical protective effect of multimorbidity that can be explained by greater engagement with the healthcare system. Patients with multiple chronic conditions often have more frequent contact with providers and closer monitoring, which reduces the onset of additional conditions, complication risks, and healthcare utilization. Consequently, patients with ≥3 comorbidities may paradoxically be less likely to require ICU admission than those with only 1–2 comorbidities, as the severity of their illness may be lower and better controlled through more consistent care engagement [26]. Another possibility is that elderly patients with many comorbidities may not be deemed to benefit from ICU care and instead get supportive care in a regular hospital room.

Drug use was also independently associated with ICU admission, reaffirming the critical role of intravenous drug use in driving not only IE incidence but also its clinical severity and cost burden [27,28]. Over half of the admitted patients with BLIE were on medications for opioid use disorder (MOUD) prior to hospitalization—a significantly higher proportion than those with RSIE (32.5%) or LSIE (20.2%)—suggesting prior health system contact but potentially inadequate engagement in continuous care. Despite this, MOUD was continued or initiated during hospitalization in less than 30% of patients with BLIE and even fewer among LSIE cases (12.7%), highlighting a critical gap in harm reduction efforts. Untreated SUD predisposes these patients towards severe complications requiring longer hospitalizations, expensive and intensive interventions, more cardiac surgeries, worse prognosis, and frequent readmission to the hospital for recurrent infections [29] These findings highlight the importance of integrating addiction screening and treatment into standard IE management. Addressing the infection without treating the underlying substance use disorder neglects a critical driver of poor outcomes. An “Endocarditis team” approach providing non-judgmental, patient-centred, comprehensive care that combines medical and addiction services to the people with drug use is critical [4,30].

Importantly, patients with BLIE required the most healthcare interventions of the three groups, with more frequent ICU admissions, surgical interventions, and specialist consultations. Both BLIE and LSIE were significantly associated with ICU admissions, a metric which serves as a proxy for disease severity, compared to RSIE. This association remained significant even after adjusting for key factors such as drug use, age, causative organisms, and comorbidities. The uneven distribution of substance use, particularly among patients with LSIE, could have attenuated the observed association between infection site and ICU admission. Consequently, the association between left-sided IE and ICU admission may be stronger than reflected in the adjusted models. These findings confirm the known severity of LSIE and highlight the urgent need for identifying and managing BLIE, a condition that is often underrecognized in the literature [31]. One possible reason for this under-recognition is the overlap between the features of BLIE, RSIE, and LSIE, which may lead to underdiagnosis [15,31].

BLIE represents a more disseminated form of IE with one of two most likely mechanisms leading to its development in the patient: (1) repeated introduction of nonsterile material into the systemic circulation (e.g., by injection drug use) or (2) the presence of intracardiac or intrapulmonary shunting, wherein infective material from a pre-existing lesion embolizes and is shunted into the opposite side of the heart, infecting a separate valve [32]. Medical comorbidities like type 2 diabetes or chronic kidney disease are known to impair functioning of the immune system, predisposing patients with persistent bloodstream seeding of pathogenic organisms to bilateral valvular disease.

Although several case reports and small studies have documented its occurrence, BLIE is not currently classified as a distinct clinical category in major guidelines from the American Heart Association [33] or the European Society of Cardiology [34]. These guidelines typically categorize infective endocarditis (IE) based on valve type (native vs. prosthetic), device involvement, and valvular involvement (RSIE vs. LSIE) [33–35]. This lack of standardized recognition has contributed to limited epidemiological data on BLIE. However, emerging evidence, including findings from our study, suggests that BLIE is associated with substantial adverse health outcomes and significant economic burden on health resources, perhaps worse than either LSIE or RSIE individually. Previous studies on multivalvular endocarditis have reported increased hemodynamic instability, higher in-hospital mortality rates, and more complex clinical management compared to single-valve disease [20,36]. Given this cumulative burden, there is a strong rationale for formally recognizing BLIE as a distinct, high-risk clinical entity in future guidelines, epidemiological research, and public health surveillance efforts.

From a systems perspective, these findings underscore the need for prevention and early diagnosis. The economic and human costs of advanced IE—particularly in BLIE—are considerable. Community-based interventions play a vital role in mitigating these challenges. Evidence has shown that harm reduction service programs such as syringe exchange initiatives, which provide clean needles and syringes, alongside educational efforts on safer injection practices, can significantly reduce the risk of infection [4,37]. Additionally, supervised injection facilities offer a controlled environment where individuals can use drugs safely, reducing the likelihood of overdose and transmission of infections. Early initiation of MOUD is equally important, as it helps individuals manage their addiction effectively and decrease the risk of associated health complications [38]. In rural regions like Appalachia, where the prevalence of drug use and the burden of IE are disproportionately high, it becomes essential to integrate these harm reduction strategies into broader public health approaches. By doing so, we can create a more comprehensive framework that not only addresses the immediate health concerns but also promotes long-term recovery and well-being for individuals in these communities. Beyond harm reduction, prevention initiatives targeting substance use itself are equally critical, especially community-based prevention programs—such as school- and family-centered interventions, peer support networks, and community campaigns promoting awareness [39]. Strengthening these upstream efforts, in tandem with harm reduction and treatment services, can create a continuum of care that addresses both the behavioral roots and medical consequences of injection-related infections.

Our study has several limitations. As a retrospective analysis, findings depend on the accuracy of documentation and may be affected by missing or incomplete data. Additionally, electronic health records do not capture factors such as housing instability, incarceration, addiction severity, and socioeconomic deprivation, which may contribute to both disease severity and healthcare utilization. These unmeasured factors could have introduced residual confounding and influenced the observed associations. Our study cohort, drawn from university-affiliated hospitals in Appalachia, may limit generalizability to other populations. Lastly, data needed to be suppressed to protect patient privacy. Nonetheless, including BLIE as a distinct group, combined with comprehensive statewide data from a large sample of patients from several centres adds important value to the IE literature. Another strength of our study is that the dataset was developed by conducting detailed chart reviews to gather granular clinical details of the patients rather than relying solely on ICD codes.

In conclusion, our findings emphasize that BLIE represents a clinically and behaviorally distinct IE subgroup characterized by dual vulnerabilities: high-risk behaviors associated with RSIE, and medical complexity typically seen in LSIE. Notably, patients with BLIE experienced a greater clinical burden, requiring more frequent specialist consultations, increased use of pain management services, higher rate of surgical interventions, and higher mortality. Our findings underscore the importance of including BLIE as a distinct category in future epidemiological and clinical investigations of valvular involvement in IE.

### Ethical approval and consent to participate

West Virginia University Institutional Review Board (IRB protocol number: 1811373348) approved this study.
